# Whipple's disease: the great masquerader—a high level of suspicion is the key to diagnosis

**DOI:** 10.1186/s12876-021-01664-1

**Published:** 2021-03-20

**Authors:** Nikolaos Melas, Rasjan Amin, Paula Gyllemark, Amil Haji Younes, Sven Almer

**Affiliations:** 1 Department of Gastroenterology and Emergency Care, Gävle Hospital, Gävle, Sweden; 2 Department of Anesthesiology and Intensive Care Unit, S:T Göran Hospital, Stockholm, Sweden; 3 Department of Infectious Diseases, Region Jönköping County, Jönköping, Sweden; 4 Division of Inflammation and Infection, Department of Biomedical and Clinical Sciences, Linköping University, Linköping, Sweden; 5 Department of Medicine, Karolinska Institutet, Solna, Sweden; 6 Division of Gastroenterology, Karolinska University Hospital, Stockholm, Sweden

**Keywords:** Whipple’s disease, *Tropheryma whipplei*, Arthritis, Gastrointestinal symptoms

## Abstract

**Background:**

Whipple's disease is a chronic infectious disease that primarily affects the small intestine, but several organs can simultaneously be involved. The disease is caused by a gram-positive bacterium called *Tropheryma whipplei*. The disease is difficult to suspect because it is rare with unspecific and long-term symptoms; it can be lethal if not properly treated.

**Case presentation:**

We here present three patients who presented with a plethora of symptoms, mainly long-standing seronegative arthritis and gastrointestinal symptoms in the form of diarrhea with blood, weight loss, fever, and lymphadenopathy. They were after extensive investigations diagnosed with Whipple's disease, in two of them as long as 8 years after the first occurrence of joint manifestations. The diagnosis was made by PCR targeting the *T. whipplei* 16S rRNA gene from small bowel specimen in all three patients, and, besides from histopathologic findings from the duodenum and distal ileum in one and mesenteric lymph nodes in another patient.

**Conclusions:**

This report aims to raise awareness of a very rare disease that presents with a combination of symptoms mimicking other and significantly more common diseases.

## Background

Whipple’s disease (WD) is an infectious disease caused by the gram-positive bacterium *Tropheryma whipplei*. The first case was presented in 1907 by the pathologist George Hoyt Whipple [1] who named the disease lipodystrophy based on storage of fat in the intestinal wall and lymph nodes found at autopsy of a 36-year-old physician who died after chronic joint problems, diarrhea and weight loss [2]. An infectious etiology was first discovered in 1961 by electron microscopy [3]. In 1991, Wilson et al. used polymerase chain reaction (PCR) which classified the bacterium within *Actinomyces* [4]. It was not until 2000 that isolation and cultivation of a strain of *T. whipplei* were reported from the heart valve of a patient with blood culture-negative endocarditis [5].

There are few studies on the prevalence and incidence of the disease. In a recent study from Italy, the prevalence was estimated to be 3 per 1,000,000 people [6]. Most cases are reported from North America and Europe. According to one study of 664 patients from the United States, 86% of cases occurred in men, 98% were of Caucasian origin, with an average age at diagnosis of 49 years [7]. Most of the patients were farmers with occupational exposure to soil or animals. In a German study, the percentage of women was higher, 22%, with a median age of 51 at the time of diagnosis [8].

The bacterium has been isolated in water, indicating that a non-fecal pathway of infection is presumed [5]. After contact with the bacterium, humans can become asymptomatic carriers of *T. whipplei* [9]. A genetic predisposition is often required to develop the disease, especially in CD11b-circulating lymphocytes which play a vital role in activating macrophages that phagocyte *T. whipplei* [9]. A link exists between the presence of HLA-B27 and the disease course [10].

The disease primarily affects the small intestine but several other organs may be involved, e.g. joints, brain, lungs, and heart. Whipple’s disease can be lethal if not treated. The natural course of classic WD is characterized by two stages. In a first, often long-lasting prodromal stage, three-quarters of patients experience non-specific symptoms, where arthritis is the first and most common manifestation [11]. In a second stage, the symptomatology is dominated by gastrointestinal manifestations, such as weight loss, nausea, and diarrhea. WD is therefore difficult to diagnose since it often has atypical manifestations and lymphadenopathy that lead to suspicion of lymphoma [12, 13]. The diagnosis is based on histopathological examination of biopsies from the small bowel and PCR analysis of the *16S* rRNA gene for *T. whipplei* [14].

Here, we report three patients with a delay of several years between the emergence of symptoms and the final diagnosis, aiming to raise the awareness of this unusual disease presenting with a combination of symptoms most often seen in far more common diseases and thereby these could be masqueraded.

## Case presentation

### Patient 1

The patient was a 37-year-old Caucasian male, sporadic smoker, with moderate alcohol consumption, worked as a truck driver. Rheumatic symptoms had started 3 years before admission to hospital, with a transient swelling of one ring finger followed by intermittent episodes of fever with symmetrical and migratory pain, stiffness and in periods, swelling of several other joints. He was treated with non-steroidal and steroidal anti-inflammatory therapy due to palindromic rheumatism (PR) without improvement. The diagnosis was changed to seronegative rheumatoid arthritis (RA) and he was treated with methotrexate plus hydroxychloroquine. The joint stiffness continued while the swelling subsided.

Nine months later, he experienced gastrointestinal symptoms; flatulence, non-bloody diarrhea, sporadic vomiting, intermittent fever and slight weight loss. The body mass index (BMI) was 18.4 kg/m^2^ (initial 19.7 kg/m^2^). Diarrhea gradually worsened with looser stool consistency, more frequent bowel emptying and abdominal pain. Anemia was detected with hemoglobin (Hb) of 120 g/L (normal range 134–170 g/L), hypoalbuminemia of 21 g/L (normal range 35–45 g/L). Stools were positive for hemoglobin and with an elevated level of calprotectin, 244 mg/kg (normal range 0–50 mg/kg). Anti-transglutaminase antibodies were negative. The symptoms aggravated with water-thin diarrhea up to 30 times a day, the onset of fatigue, and B-symptoms such as night sweats and fever peaks up to 40 degrees Celsius. A rectoscopy one month later was without pathological findings. By then, he had lost another 5 kg in weight (BMI 17 kg/m^2^) and was hospitalized. He underwent an abdominal computed tomography (CT) scan that showed signs of small bowel inflammation, why inflammatory bowel disease (IBD)—primarily Crohn’s disease—was suspected. He was given methotrexate 20 mg once a week and hydroxychloroquine 200 mg every morning. Laboratory examinations showed leukocytes 9.2 × 10^9^/L, Hb 116 g/L, reticulocytes 24.6 × 10^9^/L, platelet count 397 × 10^9^/L, C-reactive protein (CRP) 19 mg/L, albumin 22 g/L and Erythrocyte Sedimentation Rate (ESR) 16 mm/h. Fecal cultures for *Salmonella, Shigella, Yersinia,* and *Campylobacter* were negative as was cytotoxin testing for *Clostridium difficile*. A second CT scan showed pathologically enlarged lymph nodes extending paraaortically from upper abdomen down into the small pelvis and also ventrally in the mesentery. The working diagnosis was changed from IBD to malignant lymphoma and he was discharged from hospital and referred to oncological care. His condition deteriorated with significant fatigue, nausea, vomiting, and continued water-thin diarrhea 15–20 times per day with blood. He lost another 5 kg by weight (BMI 15.9 kg/m^2^).

**Fig. 1:**
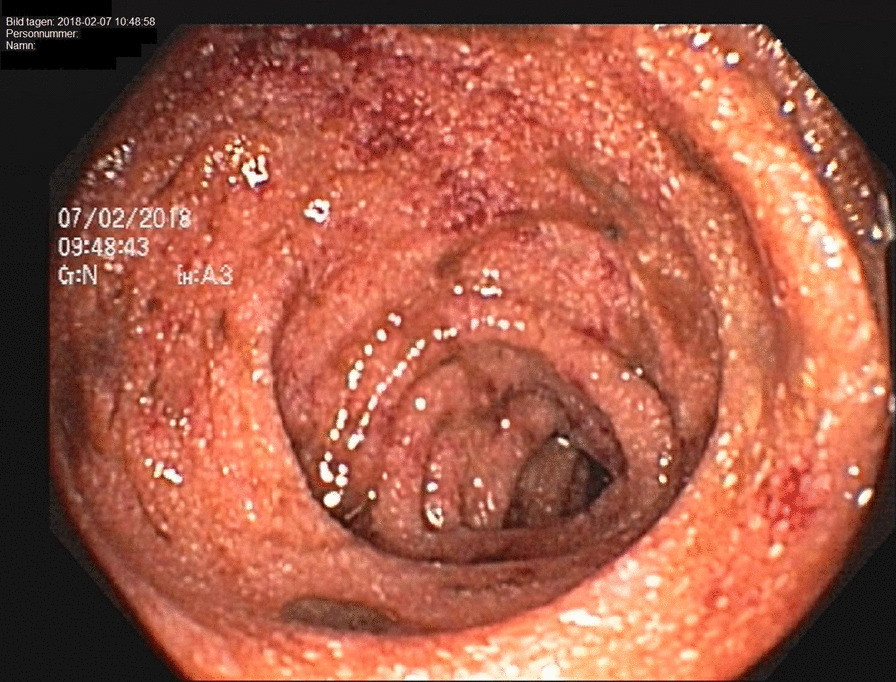
Colonoscopy. Distal ileum. Lumpy white-colored villi (see also histopathological Figs. 5 and 6) caused by ectatic lymphatic vessels that are histologically usually infiltrated by PAS-positive histiocytes. Patient 1

**Fig. 2:**
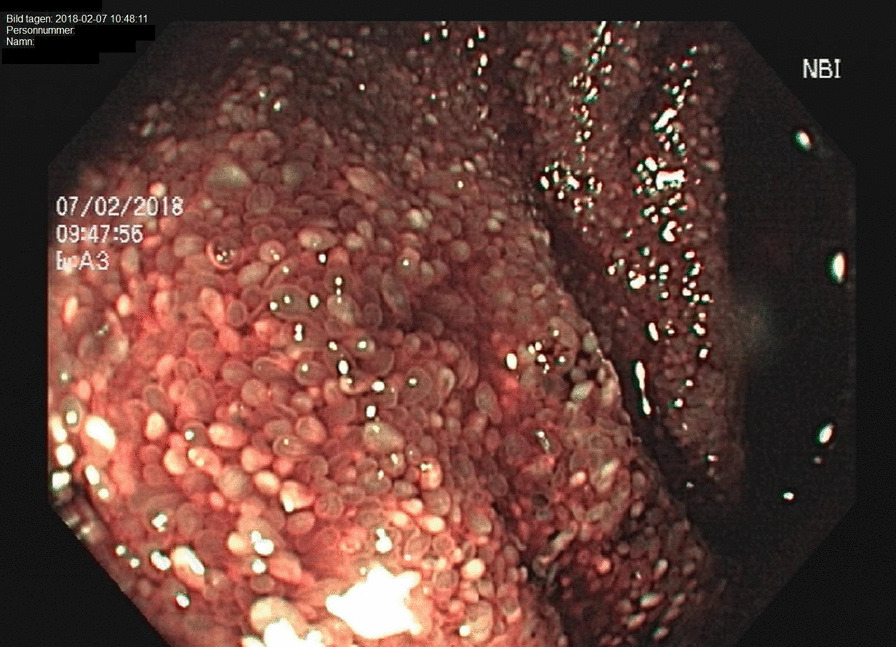
Colonoscopy. Distal ileum. See text for Fig. 1

**Fig. 3:**
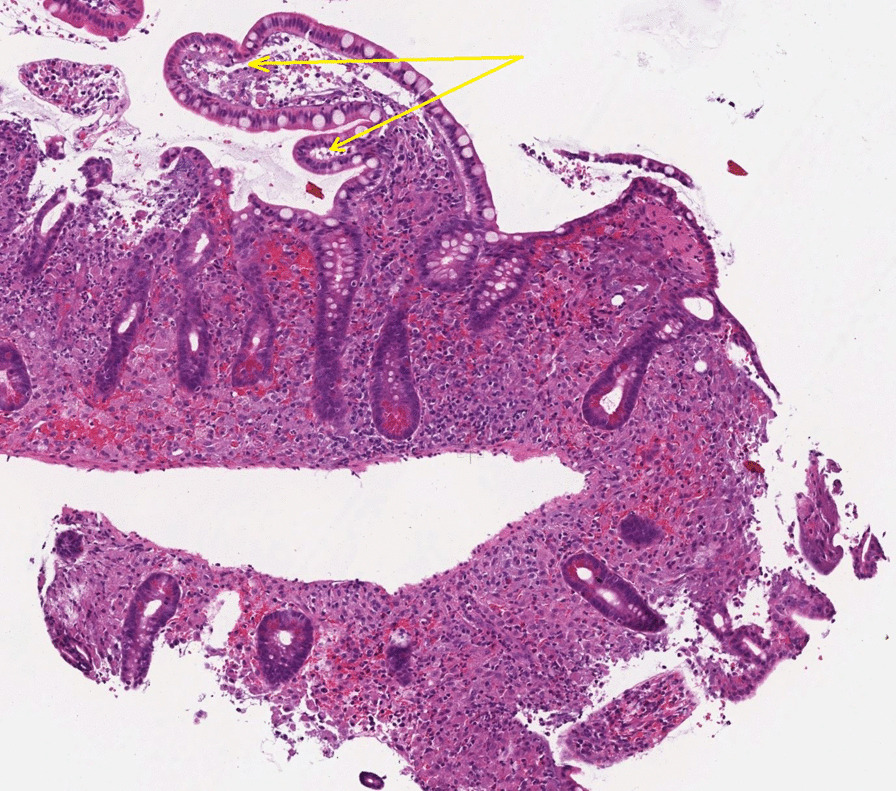
Colonoscopy. Ileum. Clearly dilated lymphatic vessels in the villi (see also endoscopic Figs. 1 and 2) that make them white. Hematoxylin–eosin staining. 11x. Patient 1

**Fig. 4:**
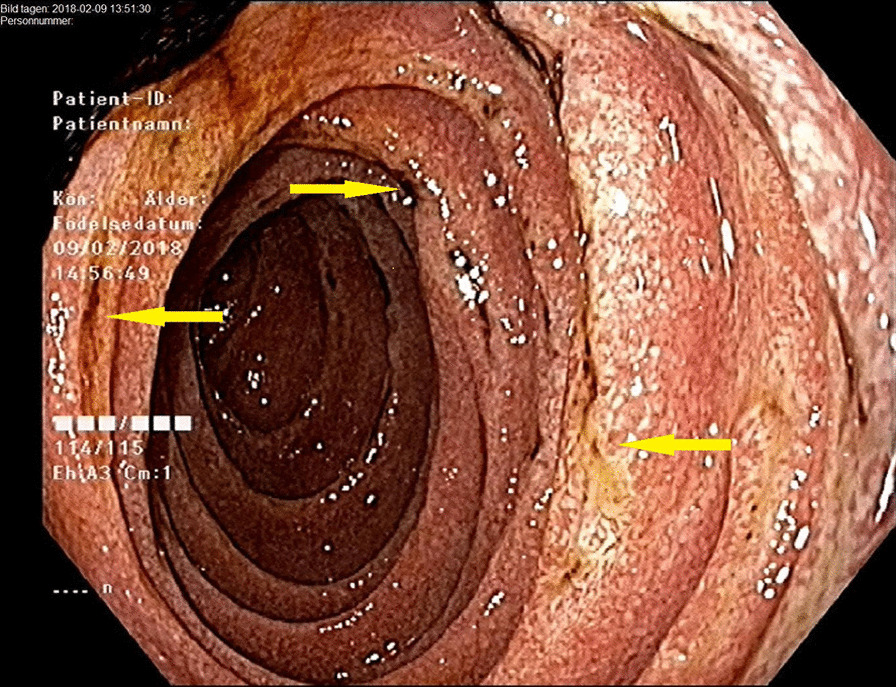
Gastroscopy. Duodenum. Diffuse mucosal redness with white-colored villi, especially abundant on folds where there are also longitudinal fibrin and hematine-coated ulcerations. Patient 1

**Fig. 5:**
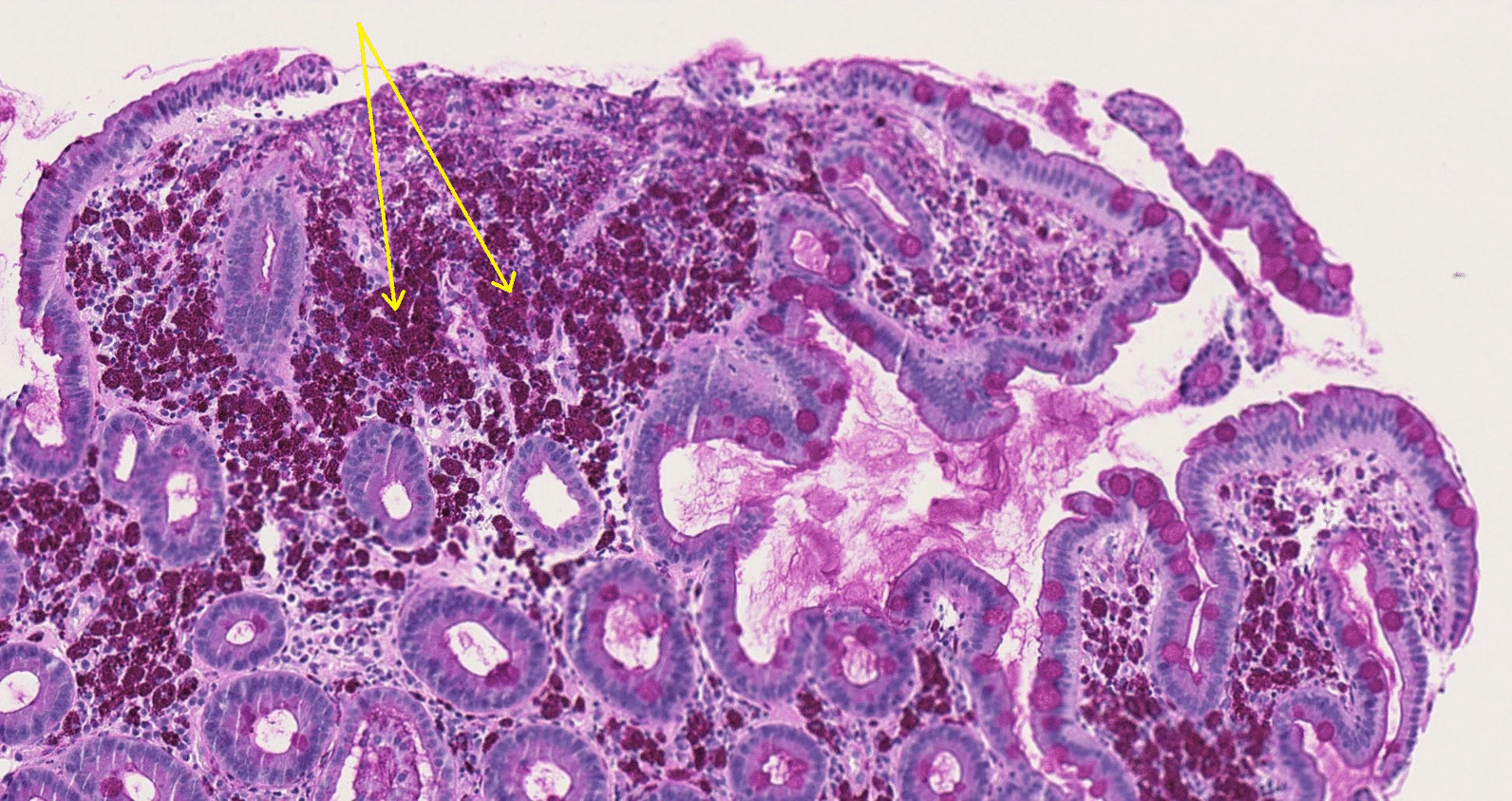
Gastroscopy. Duodenum. In lamina propria, histiocytes with PAS (periodic acid-Shiff)-positive, diastase-resistant, granulated cytoplasm is seen. 11x. Patient 1

The patient was again admitted and a colonoscopy performed, showing signs of relatively fresh bleeding and diffuse mucosal edema. Neither any evident bleeding source nor any signs of IBD were found. A diffusely swollen mucosa was seen in the distal ileum with abundant white-colored villi (Figs. 1, 2), which raised suspicion of WD. On histology of biopsies from ileum, light to moderate lymphoplasmatic cellular Periodic Acid-Schiff (PAS)-positive infiltrate was present in the lamina propria with an abundance of histiocytes; hence, a picture compatible with WD (Fig. 3). PCR for the *16S* rRNA gene of *T. whipplei* from ileum was positive. Upper endoscopy revealed acute duodenitis with abundant narrow fibrin (Fig. 4). Duodenal biopsies showed similar findings as in the ileum, i.e. PAS-positive histiocytes with diastase-resistant granulated cytoplasm diagnostic for WD (Fig. 5) and positive PCR for the *16S* rRNA gene of *T. whipplei*. Microscopy of an extirpated paraaortal lymph node was compatible with WD. Further PCR analyses for the *16S* rRNA gene of *T. whipplei* were positive in serum but negative in the cerebrospinal fluid (CSF). Transthoracic echocardiography (TTE) showed no signs of endocarditis or other abnormalities.

The patient was treated with ceftriaxone 2 g intravenously (IV) daily for two weeks with marked improvement already after three days. The diarrhea decreased, he began to support himself orally and gained 3.5 kg in weight. He was thereafter started on oral trimethoprim-sulfamethoxazole (TMP-SMX) 160 mg/800 mg twice a day for 1 year.

Three weeks after discharge from the hospital, the patient had gained an additional 3 kg with a BMI 15.8 kg/m^2^. He was still weak, became easily fatigued, and was powerless and subfebrile. He had iron deficiency anemia and pronounced zinc and vitamin D deficiencies secondary to malabsorption and received substitution. At 6 weeks after discharge, he was asymptomatic and had gained 7 kg in weight but was still slightly underweight with BMI 17.6 kg/m^2^. Six months after diagnosis, all laboratory values were normal; he had gained an additional 5 kg to BMI 18.9 kg/m^2^ and had taken up full-time work.

One and a half years after diagnosis, the patient was in good shape, had no symptoms, had normal weight and BMI. Upper endoscopy with duodenal biopsy was performed; histology was now normal and PCR for *16S* rRNA was negative for *T. whipplei*. The treatment with TMP-SMX was discontinued after altogether 18 months.

### Patient 2

A woman of Caucasian origin born in 1950, with a medical history of nodular goiter, underwent thyroidectomy in 2002 and was given levothyroxine. She had been a regular smoker until 1990, and later more sporadically. In 2007 she was seen in primary care because of morning joint pain in her left hand which she had had from time to time during 8 years. The joint pain worsened and now also engaged the shoulders, right hip, and the back. Symmetrical swelling of several metacarpophalangeal joints, of both wrists and knees was present. Repeated X-rays and magnetic resonance imaging (MRI) showed bursitis in the right hip and osteonecrosis. She was seen by many physicians and was given a diagnosis of PR, treated with hydroxychloroquine. Still, she experienced relapses and was trouble-free for a maximum of 2 weeks in-between. She had altogether tried eight anti-rheumatic drugs, however, all in suboptimal doses due to side effects or with insufficient effect. Her weight remained stable around 60 kg (BMI 22.9) until 2011 when she began to lose weight due to loss of appetite, malaise and gastrointestinal discomfort.

In June 2014, her condition deteriorated with symptoms of food aversion, night sweats, and fever. She lost a total of 12 kg (BMI 18.3) over 1 year. She was under treatment with methotrexate, and, since February 2013, in combination with adalimumab. A work-up with upper endoscopy showed gastritis and normal duodenal mucosa with unremarkable histology. Anti-transglutaminase antibodies were negative. Colonoscopy showed sigmoid diverticulosis but nothing that could explain the symptoms.

Malignant lymphoma was suspected and an abdominal CT-scan showed panniculitis. Bone marrow examination was without any signs of lymphoma. In November 2014, a new rheumatologic assessment considered her to be low active in her RA. Blood tests showed elevated CRP, ESR, and leukocytes, and, when treatment was intensified, the fever came back. Serological analyses regarding HIV, hepatitis B and C, Epstein-Barr virus (EBV), cytomegalovirus (CMV), parvovirus B19, and tick-borne encephalitis virus were all negative. MRI of the small bowel confirmed two cm-large mesenterial lymph nodes related to panniculitis. Gynecological examination and mammography were unremarkable. Tumor markers CA19-9, CA15-3, and CEA were within normal limits, CA-125 was slightly elevated. A Positron Emission Tomography-Computed Tomography (PET-CT) could not explain the increased inflammatory burden or the new symptoms.

In June 2015 the patient was referred for a second opinion. A new upper endoscopy was performed with biopsy from the duodenum. On histology, infiltration of polymorph nuclear granulocytes into lamina propria was seen along with mild cryptitis. PAS staining was negative without aberrant macrophages or any other findings compatible with WD. PCR for *T. whipplei* was positive.

Treatment started in October 2015 with ceftriaxone 2 g IV once a day for 14 days followed by TMP-SMX 800/160 mg twice a day. After 4 weeks abdominal and joint symptoms had disappeared and weight was regained. The sequential biologic therapy (infliximab, abatacept, and adalimumab) for 5.5 years was stopped. At three months the patient had normalized leukocytes, CRP, and ESR. Methotrexate treatment for 6 years was then stopped, and, within nine months after the start of antibiotics, 8 years of more or less continuous prednisolone use was no longer needed.

During the following year, she suffered periods of scleritis, necessitating courses of prednisolone and localized joint pain with the need for local steroid injections in the shoulders, in the left acromioclavicular joint, or in the right hip where an x-ray was compatible with arthrosis. Due to nausea and bowel upset, TMP-SMX was replaced by doxycycline after six months. After 13 months of antibiotic treatment, variable abdominal symptoms appeared which led to an upper endoscopy due to suspicion of disease relapse. The gastric and duodenal mucosa was normal without PAS-positive cells and negative PCR for *T. whipplei*. A CT scan after 2 years of antibiotics showed marked regression of the abdominal lymph nodes. Doxycycline was stopped at the end of December 2017 after altogether 27 months of antibiotic treatment.

A follow-up investigation at 3 years after diagnosis including testing for fecal blood and microbes, and upper endoscopy with biopsy, was negative. Repeated tests for fecal blood and calprotectin between 2015 and 2020 have all been negative. Five years after diagnosis (September 2020) the patient is doing well, she has only mild, non-specific abdominal and joint symptoms, and persisting mild polyneuropathy in feet and ankles.

### Patient 3

A 52-year-old Caucasian male was in the summer of 2018 referred to hospital with a 2-year history of 12 kg weight loss, recurrent low-grade fever, night sweats, and fatigue. He worked as an industrial worker, and had a history of regular visits to Brazil, mostly for sunbathing. Previous medical history included lower back pain with a herniated lumbar disc.

A CT-scan of the thorax and abdomen showed multiple para-aortic enlarged lymph nodes and borderline splenomegaly. The ascending aorta was slightly dilated which was confirmed by a TTE. His back pain was worsening. Laboratory results showed ESR 40 mm/h, CRP 40 mg/L, albumin 35 g/L, and Hb 130 g/L. Serology regarding EBV and CMV was positive for IgG, i.e. reflecting past infection. Serology for HIV, Q fever (*Coxiella burnettii*), *Leishmania*, *Brucella*, *Bartonella henselae*, *Bartonella quintana,* and *Trypanosoma cruzi* were all negative as was an interferon-gamma release assay and two sets of aerobic and anaerobic blood cultures.

The investigation could not find any evidence of infectious disease. A bone marrow biopsy showed signs of reactive cells but no malignant cells. A CT-guided medium needle biopsy on the para-aortic lymph nodes excluded malignancy and displayed only increased numbers of B and T lymphocytes. Immunohistochemical analysis showed numerous CD68 positive cells. MRI of the vertebral column showed an unchanged slightly herniated disc.

**Fig. 6:**
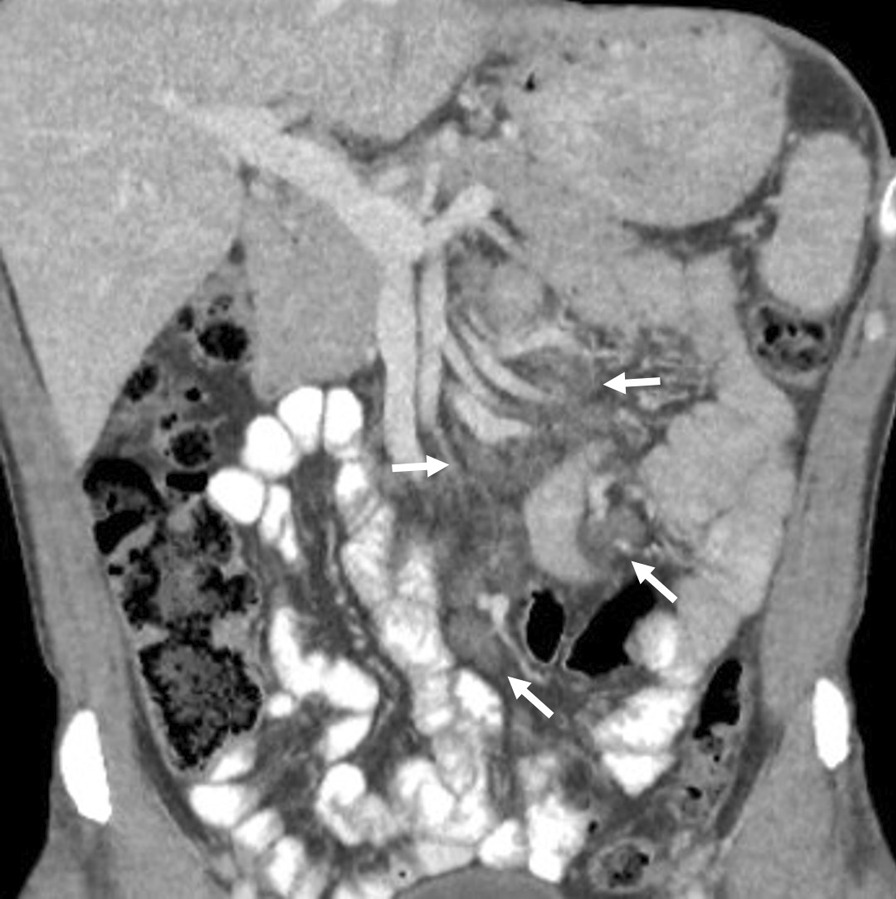
Coronal CT showing enlarged lymph nodes in the small bowel mesentery (white arrows). All of them have very low attenuation suggesting fat content which is common in Whipple’s disease, Patient 3

Symptoms remained unaltered and more than 1 year later, a new CT of the abdomen showed increased retroperitoneal lymph node enlargement (Fig. 6). At abdominal laparoscopy, three intact lymph nodes were extracted. Numerous lymphocytes, as well as many giant lymphocytes and macrophages, were found, the latter with positive PAS staining. The patient underwent upper endoscopy where the mucosa was normal and routine staining of duodenal biopsies was normal and PCR for *T. whipplei* was positive.

The patient was treated for 2 weeks with ceftriaxone (2 g IV once a day) followed by TMP-SMX 160 mg/800 mg, twice a day, planned for 1 year. Symptoms gradually disappeared within a couple of weeks, including the low back pain. After four months of treatment ESR, CRP, and Hb had normalized. No drug-induced side effects have occurred during 12 months of treatment.

## Discussion and conclusions

We report three patients with WD, with a delay of several years between the appearance of unspecific, mainly rheumatic, symptoms, and the final diagnosis. The classic onset symptom is joint discomfort in 80% of patients which precedes other symptoms. Predominantly large joints (knees, wrists, and ankles) are affected with migratory arthralgia/arthritis. Many patients are initially diagnosed with seronegative RA, and, according to a French study, half of them receive immunomodulatory therapy, including anti-TNF-α [16]. It is reported that antirheumatic drugs can worsen the course of the disease with gastrointestinal symptoms, prompting investigation with endoscopy as demonstrated by the disease evolution to patient two, with the appearance of abdominal symptoms during the year following the addition of adalimumab treatment [17].

Several characteristic similarities were seen in Patients one and two. They both had very long arthritic phases before obvious gastrointestinal symptoms appeared and led to the correct diagnosis. They were initially diagnosed as PR and had been on long-term corticosteroid and immunosuppressive therapy. Treatment with anti-TNF-α can paradoxically reveal and/or aggravate the disease, especially etanercept (Patient two), or in combination with other disease-modifying antirheumatic drugs, usually methotrexate and corticosteroids [18]. Although Patient three had symptoms of a more systemic nature and did not present joint symptoms with the need for immunosuppressive therapy, there are still similarities between all three patients. They had laboratory findings with unexplained anemia, leucocytosis, hypoalbuminemia, plus enlarged mesenterial lymph nodes, and B-symptoms with a strong suspicion of lymphoma [16], which prompted further investigations with bone marrow biopsy and lymph node extirpation. Since WD affects different organ systems, and present several more or less unspecific symptoms [15], we want to underline the similarities of our patients as the disease is truly a masquerader where a high level of suspicion is the key to early (Patient three) or delayed diagnosis (Patient one and two).

The gastrointestinal symptoms usually appear late in the disease course with intermittent water-thin diarrhea that can become bloody, cramp-like abdominal pain, and weight loss. Fever is reported in 25–40% of cases [19]. Half of the patients have lymphadenopathy, especially mesenteric and mediastinal lymph nodes, which raise suspicion of lymphoma [16]. Isolated central nervous system (CNS) symptoms, such as dementia and findings such as supranuclear ophthalmoplegia, nystagmus, or myoclonia, may also occur late. The most common cardiac manifestations in WD are endocarditis and valvular disease, seen in about half of the patients. In particular, the aortic and mitral valves are affected, but pericarditis, myocarditis, and coronary artery disease have also been reported [20]. Patient three had a mild aortic insufficiency according to TTE but the link to WD was not straightforward, since it could also be of a hereditary origin in this patient.

WD is probably an under-diagnosed disease, since the bacterium *T. whipplei* can be found in asymptomatic carriers [21], and PCR-testing can detect the *16S* rRNA of *T. whipplei* in healthy subjects [22]. The disease has a higher prevalence among middle-aged men of European origin [5]. In one study of healthy blood donors, one of 174 blood samples (0.6%) hade positive PCR for *T. whipplei* [23], and, in another study of 620 healthy individuals, PCR was positive in 0.6% of saliva samples and in 1.5% of stool samples [22].

Since most carriers of *T. whipplei* do not develop WD it is important to first rule out more common diseases with similar symptoms. These include inflammatory bowel disease, chronic infectious enterocolitis, rheumatic diseases, lymphoma, HIV infection, and tuberculosis.

Cultivation of *T. whipplei* is time-consuming, maybe false negative, and is therefore not used in clinical routine. Besides, serology is not useful for diagnosis since both patients with WD and up to 70% of healthy individuals can develop a *T. whipplei* specific humoral immune response [24]. The diagnosis of WD is therefore made histopathologically with the detection of PAS-positive inclusions in histiocytes/macrophages in the lamina propria from small intestinal mucosal biopsies, but also other tissues such as articular fluid, lymph nodes, CSF and, heart valves [14]. Direct visualization of living or dead bacteria is possible with electron microscopy [3, 25], however, not available in many centers and, nowadays rarely needed when 16sRNA testing has become available. Treatment with anti-TNF-inhibitors can abolish these ‘hallmark’ histopathological findings [18], as seen in Patient two where a first upper endoscopy missed the diagnosis, thus underlying the importance of including PCR-testing on bioptic material in any case of suspected WD.

In our first two patients, the diagnosis was primarily made through biopsy from the small bowel. Specific PCR for *T. whipplei* from saliva and stool samples has been considered to be first-line non-invasive detection of WD with high specificity if both samples are positive, but low sensitivity in localized WD where biopsy from affected tissues are needed [26]. In the majority of cases, the small intestine is involved but WD-related lesions can be minimal with false-negative results, especially when gastrointestinal symptoms are lacking. Therefore, repeated biopsies are often required for diagnosis [27]. To ensure the diagnosis of WD in these cases, two different diagnostic methods should be used such as biopsy with PAS-staining and PCR for *16S* rRNA specific for *T. whipplei*:

- Both biopsy and PCR are positive from the same samples, or
- Both biopsy and PCR are positive from different samples; e.g. positive PAS staining from the duodenum and PCR from synovial fluid, or
- Positive PCR from two different organs, e.g. both duodenum and synovial fluid.

WD has a dismal prognosis if left untreated [22]. It is important to treat with antibiotics that penetrate the blood–brain barrier given that *T. whipplei* often is present in the CSF [28]. WD without CNS involvement is initially treated with intravenous ceftriaxone 2 g once daily or meropenem (three doses of 1 g/day) for 2 weeks, followed by maintenance therapy with oral TMP-SMX 160 mg/800 mg twice a day for 1 year [29]. The treatment for WD with endocarditis is penicillin G (2 million IU IV every 4 h) or ceftriaxone (2 g IV once daily) for 4 weeks, followed by TMP-SMX 160 mg/800 mg twice a day for 1 year. CNS involvement is treated with ceftriaxone (2 g IV once daily) or penicillin G (4 million IU IV every 4 h) for 2 weeks, followed by TMP-SMX 160 mg/800 mg twice a day for 1 year. Alternative therapies in case of intolerance are meropenem (1 g IV every 8 h) for 2 weeks followed by doxycycline (200 mg a day) in combination with hydroxychloroquine (600 mg a day) for 1 year [9].

PCR for *T. whipplei* becomes negative soon after antibiotic therapy has commenced. Before stopping antibiotic therapy, upper endoscopy with duodenal biopsy and PCR testing is recommended to ensure complete remission [9].

After discontinuation of antibiotic therapy, there is a 9–15% risk of *T. whipplei* to persist latently in the body for many years, mainly in patients with CNS involvement [30]. The average interval between the end of antibiotic treatment and an ensuing relapse is 4.2 years but may occur even after 30 years [31]. Therefore, annual check-ups are recommended for at least some years after the end of treatment.

This report aims to highlight that patients with this rare condition may present at different departments with a multitude of complaints, and therefore a high level of suspicion is warranted, since today’s diagnostic work-up with upper endoscopy plus duodenal biopsy including PCR-testing is convenient for the patient, and in many of them most probably can obviate more laborious investigations.

## Data Availability

Data sharing is not applicable to this article as no datasets were generated or analysed during the current study.

## Abbreviations

BMI: Body mass index
CNS: Central nervous system
CMV: Cytomegalovirus
CRP: C Reactive protein
CSF: Cerebrospinal fluid
CT: Computed tomography
EBV: Epstein Barr virus
ESR: Erythrocyte sedimentation rate
Hb: Haemoglobin
IBD: Inflammatory bowel disease
IV: Intravenously
MRI: Magnetic resonance imaging
PAS: Periodic acid Schiff
PET: Positron emission tomography
PCR: Polymerase chain reaction
PR: Palindromic rheumatism
RA: Rheumatoid arthritis
SMX: Sulfamethoxazole
TMP: Trimethoprim
TTE: Transthoracic echocardiography
WD: Whipple’s disease
